# Pure red cell aplasia post-SARS-CoV-2 infection: a case report in an HIV-positive patient

**DOI:** 10.3389/fmed.2025.1674188

**Published:** 2025-10-20

**Authors:** Yanli Wang, Shi Tang, Renjie Bian, Ying Wen

**Affiliations:** ^1^Department of Infectious Diseases II, The First Affiliated Hospital of China Medical University, Shenyang, Liaoning, China; ^2^Department of Hematology, The First Affiliated Hospital of China Medical University, Shenyang, Liaoning, China

**Keywords:** pure red cell aplasia, SARS-CoV-2 infection, HIV-positive, AIDS, case report

## Abstract

We present a case of Pure Red Cell Aplasia (PRCA) secondary to severe acute respiratory syndrome coronavirus 2 (SARS-CoV-2) infection in a human immunodeficiency virus (HIV)-positive patient. To our knowledge, this is the first reported case of SARS-CoV-2-associated PRCA in an HIV-positive individual. The patient received immunosuppressive therapy (IST):initial treatment with corticosteroids was later switched to cyclosporine A (CsA), and he ultimately achieved complete remission. Additionally, we reviewed previously reported cases of SARS-CoV-2-associated PRCA. These findings emphasize that hematological abnormalities should also be recognized as a critical extrapulmonary complication of SARS-CoV-2 infection. In conclusion, SARS-CoV-2 infection can affect bone marrow tissue, leading to a spectrum of hematological abnormalities and subsequent clinical sequelae. For HIV-positive patients presenting with PRCA, antiretroviral drugs and acute or chronic infections should be prioritized in the diagnostic workup.

## Introduction

Severe acute respiratory syndrome coronavirus 2 (SARS-CoV-2) infection has been shown to cause multiple hematological abnormalities, which impair blood cell production and function (1). Recent studies have systematically summarized cases of SARS-CoV-2-associated aplastic anemia (AA) (2). Additionally, cases of SARS-CoV-2-associated pure red cell aplasia (PRCA) have also been reported previously (3–9). Among individuals infected with human immunodeficiency virus (HIV), anemia is common and has multifactorial causes. However, PRCA was a rare hematological abnormality in HIV-positive patients, most frequently induced by antiretroviral drugs, followed by parvovirus B19 infection. Herein, we present a case of PRCA secondary to SARS-CoV-2 infection in a HIV-positive patient. In May 2023, the patient experienced a second episode of SARS-CoV-2 infection, again presenting with fever, sore throat and cough, confirmed by a positive SARS-CoV-2 antigen test. On June 12, 2023, he presented with fatigue and dizziness. Laboratory investigations revealed a Hb level of 31 g/dL, an absolute reticulocyte count of 4,100 /μL, and a reticulocyte percentage of 0.42%. The patient received immunosuppressive therapy (IST): initial treatment with corticosteroids was later switched to cyclosporine A (CsA). Ultimately, he achieved complete remission. These findings emphasize that hematological abnormalities should also be recognized as an important extra-pulmonary complication of SARS-CoV-2 infection. This case report adheres to the requirements of the CARE case report guidelines.

## Case description

A 33-year-old Chinese male hairdresser (with 14 years of occupational experience) presented with a one-month history of fatigue and dizziness in January 2023. He had experienced his first SARS-CoV-2 infection in December 2022, manifested as fever, sore throat and cough, which was confirmed by a positive SARS-CoV-2 antigen test. During that outpatient visit, laboratory testing revealed anemia with a hemoglobin (Hb) level of 61 g/dL, while his leukocyte and platelet counts were normal (no further diagnostic workup was performed at the time). The patient had previously received three doses of inactivated SARS-CoV-2 vaccine and achieved spontaneous complete recovery two months later, in April 2023. In May 2023, the patient developed a second SARS-CoV-2 infection, again presenting with fever, sore throat and cough, confirmed by a positive SARS-CoV-2 antigen test. On June 12, 2023, he presented with fatigue and dizziness; laboratory investigations revealed a Hb level of 31 g/dL, absolute reticulocyte count of 4,100/μL, and a reticulocyte percentage of 0.42%. At this time, SARS-CoV-2 testing by polymerase chain reaction (PCR) was negative. Other laboratory findings included a negative direct Coombs test, no evidence of a paroxysmal nocturnal hemoglobinuria (PNH) clone or nutritional anemia, a normal serum protein electrophoresis, negative rheumatologic antibodies, and undetectable serum cytomegalovirus (CMV) DNA and Epstein–Barr virus (EBV) DNA. PRCA was confirmed by bone marrow morphology, which showed severe erythroid hypoplasia (with only 0.4% proerythroblasts) but normal granulocytic and megakaryocytic maturation. Additionally, parvovirus B19 IgG/IgM antibodies and DNA were not detected in his blood. Chest computed tomography (CT) scan showed no thymus abnormalities. The patient denied a history of smoking or alcohol consumption, and no family members or coworkers had a history of similar hematological disorders. The patient had been diagnosed with HIV-1 in 2019 with a nadir CD_4_^+^ T-cell count of 66 cells/μL, and had initiated antiretroviral therapy (ART) with lamivudine, tenofovir disoproxil and efavirenz. In June 2021, he was found to have developed resistance to the above three anti-HIV drugs (mutations detected by Sanger sequence including K65R, M184MI, V75I, Y181C, G190S). In July, 2021, his ART regimen was switched to dolutegravir combined with lopinavir/litonavir due to gastrointestinal intolerance to zidovudine. Following the initiation of lopinavir/ritonavir, the patient developed worsening hyperlipidemia, particularly hypertriglyceridemia (peak level of 25.6 mmol/L). We recommended fenofibrate for the patient, but he refused. Above all, antiretroviral drugs, parvovirus B19, and hairdye were not considered as the causes of PRCA. Finally, SARS-CoV-2-associated PRCA was considered as the probably etiology of PRCA in this case.

**Figure 1:**
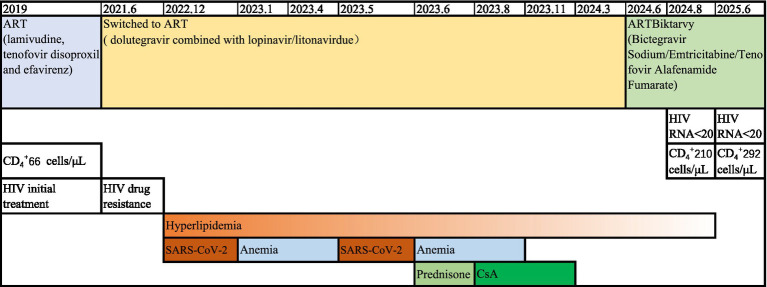
The clinical course timeline.

In contrast to the first episode, which resolved spontaneously, the second episode did not show spontaneous resolution. The patient received five times of red blood cell (RBC) transfusions. At this time, his CD_4_^+^ T-cell counts was 143 cells/μL, and HIV RNA was undetectable (<20 copies/mL). Given the potential drug–drug interaction (DDI) between CsA and lopinavir/litonavir, the patient was initiated on a regimen of 60 mg/day prednisone on June 2023. However, no improvement in the anemia was observed, as evidenced by the lack of a significant Hb and reticulocyte response. Then he initiated CsA (200 mg/d) in August 2023. We noted that lopinavir/litonavir can obviously increase the plasma concentrations of CsA, frequent concentration monitoring of CsA was performed. For the 200 mg/day dose, therapeutic drug monitoring revealed trough (C0) and 2-h post-dose (C2) CsA concentrations of 559 ng/mL and 795.6 ng/mL, respectively. The CsA dose was therefore reduced to 100 mg/day in September 2023, which resulted in stable C0 concentrations of 300 ng/mL and no CsA-related adverse events (10). He returned back to his workplace as a hairdresser. In November 2023, he achieved a complete hematologic response (CR) with a Hb level of 130 g/dL without fatigue and dizziness. CsA was discontinued in March 2024. In June 2024, his ART regimen was switched to Biktarvy (Bictegravir Sodium/Emtricitabine/Tenofovir Alafenamide Fumarate) due to uncontrolled heperlipididemia. Since then, his hypertriglyceridemia has improved (average level of 5.62 mmol/L). In August 2024, his CD_4_^+^ T-cell count had increased to 210 cells/μL, HIV RNA remained undetectable, and Hb level was within the normal range. The most recent follow-up (February 2025) confirmed continued virologic suppression (HIV RNA < 20 copies/mL), a further increase in CD4^+^ T-cell count to 292 cells/μL, and no relapse of anemia. The patient’s complete clinical course is summarized in Figure 1. A written informed consent was obtained from the patient for publication.

## Discussion

PRCA is clinically characterized by isolated anemia with normal white cell and platelet counts. Bone marrow evaluation is pathognomonic for the disorder: near-complete absence of erythroblasts (erythroid hypoplasia) is observed, accompanied by preserved maturation of myeloid cells and megakaryocytes. Etiologically, PRCA is classified into primary and acquired subtypes, with acquired PRCA accounting for the majority of cases—including the one presented herein. Acquired PRCA has been linked to multiple causative factors, such as viral infections, medications, hematologic malignancies, autoimmune diseases, and thymomas (11). Current research suggests that, except for parvovirus B19-induced transient aplastic crisis (TAC), most idiopathic cases arise from T cell-mediated destruction of erythroid progenitor cells (12–14). In contrast, drug-associated PRCA is thought to result from direct toxic effects of medications on erythroid progenitors, specifically burst-forming unit-erythroid (BFU-E) and colony-forming unit-erythroid (CFU-E) cells. In HIV-positive patients, nucleoside reverse transcriptase inhibitors (NRTIs) including zidovudine, lamivudine, and emtricitabine, have been well-documented as etiologic agents of ART-associated PRCA (15–17). NRTIs mainly showed mitochondrial toxicity. HIV-positive cases usually presented immune dysregulation, and PRCA typically develops within several months of initiating ART. Emtricitabine-induced PRCA is generally reversible, with most patients achieving hematologic recovery within 4 months of discontinuing the drug (17). However, the present case developed anemia while receiving a regimen of dolutegravir plus lopinavir/ritonavir—two antiretroviral agents with no prior reports linking them to PRCA. Another well-recognized cause of PRCA in HIV-positive individuals is persistent parvovirus B19 infection, which typically occurs in patients with severe immunodeficiency (e.g., those who have not initiated ART or have interrupted ART) (18). Parvovirus B19 can directly infects erythroid progenitor cells via the P antigen expressed on erythrocyte surfaces. A pathognomonic feature of parvovirus B19-associated PRCA is the presence of large proerythroblasts with vacuolated cytoplasm and pseudopods in bone marrow aspirates. However, serum testing for parvovirus B19 IgG/IgM antibodies and DNA were both negative. Therefore, parvovirus B19 was not considered as the cause of PRCA in this case. When the second episode of anemia, his HIV RNA was undetectable. Therefore, HIV itself as the etiology of PRCA was not considered. Other opportunistic infections such as CMV and EBV were also excluded for this case. Occupational exposure to hairdye-related chemicals was also ruled out. The patient had 14 years of experience as a hairdresser, but no coworkers reported similar hematological disorders. Furthermore, the patient resumed work as a hairdresser after achieving complete hematologic remission of PRCA and has since remained anemia-free during follow-up. Thymoma-associated PRCA was excluded based on chest computed tomography (CT) findings, which revealed no thymic abnormalities. Finally, erythropoietin-induced PRCA was excluded, as the patient had no prior history of recombinant human erythropoietin administration.

To our knowledge, this represents the first reported case of SARS-CoV-2-associated PRCA in an HIV-positive individual. The clinical characters of previously reported cases of SARS-CoV-2-associated PRCA are summarized in Table 1. A total of 9 cases have been reported, with all patients being male and aged 2–77 years. The interval between SARS-CoV-2 infection and PRCA onset ranged from 10 to 120 days. Three cases were complicated by concurrent or prior autoimmune hemolytic anemia (AIHA). One patient had a history of chronic glomerulonephritis requiring regular hemodialysis and recombinant human erythropoietin therapy; this patient tested positive for anti-erythropoietin antibodies (9). Only our case was HIV-infected case. None of the patients received effective anti-SARS-CoV-2 medications. There were 3 cases with corticosteroids and two cases with CsAs yielded favorable outcomes. Two cases showed good efficacy with tacrolimus. One patient showed a recovery only with RBC transfusion.

**Table 1:** The clinical characters of reported cases of SARS-CoV-2-associated PRCA.

First author name/year of publication	Country	Age (years)/gender	Past medical history	Progromic SARS-CoV-2 infection	Nucleic acid result of SARS-CoV-2 when PRCA occurrence	Application of effective anti-SARS-CoV-2 drugs	Clinical types of COVID-19	The duration between SARS-CoV-2 infection and PRCA occurrence	Complete blood count	Reticulocyte count (/μL)	Bone marrow cellularity	Diagnosis	Treatment AA	Outcome
Lee et al. (2022) (3)	USA	76 M	NM	Positive	NM	NM	NM	4 months	WBC: 10.3×10 9/L, Hb: 59 g/L, Platelet: 298×10 9/L	8000	NM	PRCA	CsA/tacrolimus	Recovered
Yamazaki et al. (2022) (4)	Japan	67 M	None	Positive	NM	No	Critical	24 days	Hb: 74 g/L	NM	Erythroid hypoplasia (0.5% erythroblast)	AIHA, then PRCA	CsA	Recovered
Karrat et al. (2022) (5)	Arab	77 M	Hypertension	Positive	Negative	No	Severe	8 weeks	WBC: 3.4×10 9/L, Hb: 50 g/L, Platelet: 202×10 9/L	30300	Erythroid hypoplasia (<1% erythroblast)	PRCA	Oral prednisone 1 mg/kg	Recovered
Ghimire et al. (2022) (6)	Canada	29 M	Obesity, mild asthma, and gastroesophageal reflux	Positive	NM	NM	Critical	10 days	WBC: 15.8×10 9/L, Hb: 38 g/L, Platelet: 549×10 9/L	13500	Erythroid hypoplasia (NM)	AIHA, then PRCA	Dexamethasone	Recovered
Kimura et al. (2023) (7)	Japan	28 M	AIHA for 5 years	Positive	Weakly positive	No	Mild	4 weeks	WBC: 7.59×10 9/L, Hb: 37 g/L, Platelet: 236×10 9/L	8000	Erythroid hypoplasia (1.8% erythroblast)	AIHA and PRCA (parvovirus B19 infection?)	Prednisolone (1 mg/kg)	Recovered
Rivetti et al. (2024) (8)	Italy	2 M	NM	Positive	NM	NM	Mild	2 weeks	Hb: 46 g/L	10000	Complete absence of erythroblasts and signs of erythrophagocytosis	TEC	Transfusion with 150 ml of packed red blood cells	Recovered
Xu et al. (2024) (9)	China	49 M	Chronic glomerulonephritis, Renal anemia (regular injection of erythropoietin), hypertension, Hepatitis B virus carrier	Positive	NM	No	Mild	2 months	Hb: 44 g/L	1000	Hypercellular marrow. Erythroid precursor cells were notably absent. Granulomatous hyperplasia predominated.	PRCA (anti-EPO antibodies positive)	Prednisone and CsA/tacrolimus	Recovered
Our case.	China	33 M	AIDS	Positive	Negative	No	Mild	1 month	WBC: 5.26×10 9/L, Hb: 31 g/L, Platelet: 286×10 9/L	4100	Erythroid hypoplasia (0.4% erythroblast)	PRCA	Prednisone 60mg/d/CsA (200mg/d)	Recovered

NM, not mention; CsA, cyclosporine-A; AIHA, autoimmune hemolytic anemia; PRCA, pure red cell aplasia; WBC, white blood cell; Hb, hemoglobin; M, male; TEC, transient erythroblastopenia of childhood; EPO, erythropoietin.

The exact mechanism by which SARS-CoV-2 infection induces PRCA remains unclear. Yamazaki et al. reported not only evidence of PRCA in the bone marrow but also infiltration of CD8+, perforin-positive, granzyme B-positive cytotoxic T cells. However, the relationship between these T cells and SARS-CoV-2 has not yet been established (4). Unlike parvovirus B19, which directly infects and lyses erythroid progenitors, SARS-CoV-2 is unlikely to exert a direct toxic effect on early erythroid precursors. In contrast, parvovirus B19-associated PRCA often necessitates intravenous immunoglobulin (IVIG) therapy. Therefore, we propose that SARS-CoV-2-associated PRCA is most likely mediated by a T cell-dependent immune process, consistent with the mechanism implicated in the majority of acquired PRCA cases. This also explains why IST treatment is effective and the prognosis is generally favorable in SARS-CoV-2-induced PRCA.

The first notable feature of this case is that the patient developed anemia twice following two episodes of prodromal SARS-CoV-2 infections. Some PRCA cases exhibit an acute self-limited course. CsA has demonstrated a superior overall response rate (ORR) compared to corticosteroids in PRCA treatment (12). Moreover, corticosteroids treatment showed high relapse rate (19). Tacrolimus/Sirolimus can be the option for individuals with refractory/relapsed PRCA (9, 20, 21). For HIV-positive patients with parvovirus B19-associated PRCA, a combination of IVIG and effective ART are strongly recommended (18). The second key feature of this case is HIV-positive, and the potential DDI between ART and CsA, as well as ART-related adverse effect such as severe hypertriglyceridemia should be pay more attention to this patient. In this HIV-positive patient with a history of virological failure on a regimen containing a non-nucleoside reverse transcriptase inhibitor (NNRTI) and two NRTIs, his mutation scoring of tenofovir disoproxil was 40 (being at intermediate level drug resistance), Biktarvy represents a non-inferiority alternative for second-line ART that avoids protease inhibitors in this condition (22, 23). Fortunately, emtricitabine-containing regimen of Biktarvy did not show hematologic toxicity in this patient. This study has several limitations that should be acknowledged. First, we did not serially monitor levels of anti-SARS-CoV-2 IgG and IgM antibodies. Second, no comprehensive hematological workup was performed during the patient’s first episode of anemia: critical evaluations such as reticulocyte count/percentage and bone marrow aspiration were not conducted. This was attributed to the mild, transient nature of the first anemia episode, which led the patient to underestimate the condition and decline additional testing. In contrast, the second episode of anemia was severe and persistent, prompting the patient to recognize the urgency of care and consent to a full diagnostic workup (including hematology and infectious diseases consultations). This study highlighted the acute viral infections such as SARS-CoV-2 could be an etiology of PRCA onset, and a multidisciplinary team can provide the advantages for rapid diagnosis and optimal treatment.

In conclusion, SARS-CoV-2 infection can affect the bone marrow tissue, leading to various hematological abnormalities and associated clinical consequences (24). In HIV-positive individuals presenting with PRCA, the etiologies of antiretroviral drugs and acute or chronic infections should be prioritized in the diagnostic workup.

## Data Availability

The original contributions presented in the study are included in the article/supplementary material, further inquiries can be directed to the corresponding authors.

## References

[1] Parham E Ahmad M Falasca M. Haematological manifestations of SARS-CoV-2: insights into erythropoiesis, Hepcidin regulation, and cytokine storm. Int J Mol Sci. (2025) 26:874. doi: 10.3390/ijms26030874, PMID: 39940645 PMC11817086

[2] Cahuapaza-Gutierrez NL. Aplastic Anemia in the light of the COVID-19 pandemic: infection, vaccination, and pathophysiologic mechanisms. Ann Hematol. (2024) 103:4989–5005. doi: 10.1007/s00277-024-06052-9, PMID: 39441353

[3] Lee NCJ Patel B Etra A Bat T Ibrahim IF Vusirikala M. SARS-CoV-2 infection associated with aplastic anemia and pure red cell aplasia. Blood Adv. (2022) 6:3840–3. doi: 10.1182/bloodadvances.2022007174, PMID: 35452511 PMC9040401

[4] Yamazaki S Naito E Sekiya R Yogi S Komiyama K Miyakawa Y. Pure red cell aplasia accompanied by COVID-19 successfully treated using cyclosporine. J Infect Chemother. (2022) 28:304–7. doi: 10.1016/j.jiac.2021.10.018, PMID: 34772624 PMC8542443

[5] Karrat I Eddou H. Acquired pure red cell aplasia after severe acute respiratory syndrome corona virus 2 infection: a case report. J Med Case Rep. (2022) 16:375. doi: 10.1186/s13256-022-03545-x, PMID: 36258221 PMC9579546

[6] Ghimire A Platnich J Chauhan U. Warm autoimmune hemolytic Anemia and pure red cell aplasia during a severe COVID-19 B.1.1.7 infection. Infect Dis Rep. (2022) 14:413–9. doi: 10.3390/idr14030044, PMID: 35735754 PMC9223138

[7] Kimura H Furukawa M Shiga Y Kai T Yasuda I Katoh S. Exacerbation of autoimmune hemolytic anemia associated with pure red cell aplasia after COVID-19: a case report. J Infect Chemother. (2023) 29:787–91. doi: 10.1016/j.jiac.2023.04.002, PMID: 37044274 PMC10084666

[8] Rivetti G Abbate FG Longobardi M Marrapodi MM Lanzaro F Di Martino M. Transient erythroblastopenia of childhood after COVID-19 infection: a case report. Ital J Pediatr. (2024) 50:131. doi: 10.1186/s13052-024-01700-2 39075575 PMC11288083

[9] Feng Y Liang Y Feng Z Liu S Xu L. Tacrolimus treatment of pure red cell aplasia due to anti-erythropoietin antibodies induced by COVID-19 in hemodialysis patient: a case report. Ann Clin Med Case Rep. (2024) V14:1–5.

[10] Sawada K Hirokawa M Fujishima N Teramura M Bessho M Dan K. Long-term outcome of patients with acquired primary idiopathic pure red cell aplasia receiving cyclosporine a. a nationwide cohort study in Japan for the PRCA collaborative study group. Haematologica. (2007) 92:1021–8. doi: 10.3324/haematol.11192, PMID: 17640861

[11] Means RT Jr. Pure red cell aplasia. Blood. (2016) 128:2504–9. doi: 10.1182/blood-2016-05-717140, PMID: 27881371

[12] Gurnari C Maciejewski JP. How I manage acquired pure red cell aplasia in adults. Blood. (2021) 137:2001–9. doi: 10.1182/blood.2021010898, PMID: 33657207 PMC8057257

[13] Zhang R Han B. Exploration of B- and T-cell receptor repertoires reveals distinct mechanisms in pure red cell aplasia, autoimmune hemolytic anemia, and aplastic anemia. Acta Haematol. (2025) 30:1–17. doi: 10.1159/000547027 PMC12503455 40587957

[14] Castro-Váquez A Esquivel JL Martín JL Rosner JM. Failure of stressful stimuli to inhibit embryo implantation in the rat. Am J Obstet Gynecol. (1975) 121:968–70. doi: 10.1016/0002-9378(75)90919-9, PMID: 1167735

[15] Blanche P Silberman B Barreto L Gombert B Sicard D. Reversible zidovudine-induced pure red cell aplasia. AIDS. (1999) 13:1586–7. doi: 10.1097/00002030-199908200-00023, PMID: 10465087

[16] Kakubu MAM Bikinesi T Katoto PDMC. Lamivudine induced pure red cell aplasia and HIV-1 drug resistance-associated mutations: a case report. Oxf Med Case Rep. (2023) 2023:106–109. doi: 10.1093/omcr/omad022, PMID: 36993835 PMC10041944

[17] Manickchund N du Plessis C John MA Manzini TC Gosnell BI Moosa MS. A case series of emtricitabine-induced pure red cell aplasia. South Afr J HIV Med. (2021) 22:1271. doi: 10.4102/sajhivmed.v22i1.1271, PMID: 34522429 PMC8424770

[18] Mendes-de-Almeida DP Bokel JPB Alves ADR Vizzoni AG Tavares ICF Silva MST. Clinical presentation of parvovirus B19 infection in adults living with HIV/AIDS: a case series. Viruses. (2023) 15:1124. doi: 10.3390/v15051124, PMID: 37243210 PMC10223798

[19] Sawada K Fujishima N Hirokawa M. Acquired pure red cell aplasia: updated review of treatment. Br J Haematol. (2008) 142:505–14. doi: 10.1111/j.1365-2141.2008.07216.x, PMID: 18510682 PMC2592349

[20] Jiang H Zhang H Wang Y Qi W Cao Q Xing L. Sirolimus for the treatment of multi-resistant pure red cell aplasia. Br J Haematol. (2019) 184:1055–8. doi: 10.1111/bjh.15245, PMID: 29741762

[21] Long Z Yu F Du Y Li H Chen M Zhuang J. Successful treatment of refractory/relapsed acquired pure red cell aplasia with sirolimus. Ann Hematol. (2018) 97:2047–54. doi: 10.1007/s00277-018-3431-5, PMID: 29982851

[22] Aboud M Kaplan R Lombaard J Zhang F Hidalgo JA Mamedova E. Dolutegravir versus ritonavir-boosted lopinavir both with dual nucleoside reverse transcriptase inhibitor therapy in adults with HIV-1 infection in whom first-line therapy has failed (DAWNING): an open-label, non-inferiority, phase 3b trial. Lancet Infect Dis. (2019) 19:253–64. doi: 10.1016/S1473-3099(19)30036-2, PMID: 30732940

[23] Paton NI Musaazi J Kityo C Walimbwa S Hoppe A Balyegisawa A. Efficacy and safety of dolutegravir or darunavir in combination with lamivudine plus either zidovudine or tenofovir for second-line treatment of HIV infection (NADIA): week 96 results from a prospective, multicentre, open-label, factorial, randomised, non-inferiority trial. Lancet HIV. (2022) 9:e381–93. doi: 10.1016/S2352-3018(22)00092-3, PMID: 35460601

[24] Zeylabi F Nameh Goshay Fard N Parsi A Pezeshki SMS. Bone marrow alterations in COVID-19 infection: the root of hematological problems. Curr Res Transl Med. (2023) 71:103407. doi: 10.1016/j.retram.2023.103407, PMID: 37544028

